# Visual working memory prioritization modulates serial dependence beyond simple attentional effects

**DOI:** 10.1186/s12915-025-02441-2

**Published:** 2025-11-14

**Authors:** Ekaterina Andriushchenko, Andrey Chetverikov, Gianluca Campana

**Affiliations:** 1 Department of General Psychology, University of Padova, Via Venezia 8, Padova, 35131 Italy; 2 Department of Psychosocial Science, Faculty of Psychology, University of Bergen, Christies Gate 12, Bergen, 5015 Norway; 3 Human Inspired Technology Research Centre, University of Padova, Via Venezia 8, Padova, 35131 Italy

**Keywords:** Serial dependence, Visual working memory, Visual perception, Contextual bias

## Abstract

**Background:**

Serial dependence (SD) is a contextual bias in visual processing, where current perception is influenced by past stimuli. This study explores how prioritization in visual working memory (VWM) modulates SD through three experiments.

**Results:**

Experiment 1 revealed that tasks requiring active memory maintenance (thus prioritization in VWM) amplified SD, with stronger biases observed when participants retained prior stimuli for extended periods. Conversely, Experiments 2 and 3, which employed pre- and post-cueing in a dual-stimuli setup, found no significant differences in SD strength between congruent and incongruent conditions, suggesting that simple attentional prioritization alone does not influence SD magnitude.

**Conclusions:**

The results highlight the nuanced interplay between memory maintenance, attention, and perceptual biases, suggesting that SD arises from complex interactions beyond simple attentional mechanisms. This study advances the understanding of SD within perceptual decision-making, underscoring the role of memory maintenance in shaping visual judgments.

## Background

Vision is often considered a direct reflection of the world around us. In reality, it is inherently shaped by contextual information that helps to transform a constantly fluctuating stream of stimuli into a stable and coherent visual experience [17, 40, 55]. At the same time, context might bias perception when the information it provides is irrelevant. This paper focuses on serial dependence — a pervasive contextual bias in visual information processing — and explores how prioritization in memory modulates this phenomenon, testing the predictions of the Bayesian framework [16, 55] and the Demixing Model [14].

Serial dependence (SD) refers to the tendency of the perception of a current stimulus to be biased toward previously encountered information [17, 39, 46]. While initially regarded as purely a perceptual phenomenon ([21], but see also [22, 31], subsequent studies have revealed that it can also be affected by post-perceptual processes [1, 6, 10–13, 20, 28]. Numerous studies have highlighted the role of visual working memory (VWM) in modulating the strength of SD (see [46], for a review) as well as the impact of memory reactivations in enhancing biases [5, 6, 20]. Recent research has further deepened this understanding by uncovering the direct neural signature of SD in VWM and emphasizing the role of later processing stages in VWM representations [20, 52].

We reasoned that if working memory plays a role in serial dependence, then the priority with which an item is kept in memory should affect the strength and potentially the direction of serial dependence.

### Prioritization in visual working memory

VWM is limited, and the amount of resources allocated to keep an item in memory can be manipulated by providing participants with cues about the probability that it will be tested later (see [8], for a review). Such prioritization affects both how precisely an item is remembered and the extent to which it is biased by the presence of other stimuli or influences their perception or memorization [27, 33, 42, 44, 51, 61, 64, 65].

Both behavioral and neuroimaging studies show a clear effect of prioritization on memory precision. When a cue predicts the location of the probed stimulus, error variance is reduced, showing an increased precision of VWM representations [7, 30, 63–65]. Further support for the heightened precision of prioritized representations comes from decoding studies demonstrating that prioritized memories are more stable and accessible than unprioritized ones [33, 50, 51, 53, 62]. This flexible mechanism allows memories to be prioritized without necessarily compromising other stored items [43, 61].

Prioritization in VWM also affects the magnitude of biases in the behavioral responses. Some studies suggested that prioritization can reduce the likelihood of catastrophic memory loss (fewer “swap errors”) but amplify attraction effects toward the distractors [64, 65]. Others have found that prioritization in VWM can decrease the attraction towards distractors [51]. The conflicting findings highlight the need for further investigation to clarify how prioritization influences the direction of VWM effects on biases.

When it comes to SD, several studies manipulated the priority of to-be-reported stimuli by cueing their locations or other features. In a seminal study, Fischer & Whitney [21] found that when nine stimuli appear simultaneously, only the cued one affects the perceived orientation in the subsequent trial. Fritsche & De Lange [23] and Fischer et al. ([19], Exp. 1 & 2) presented observers with a sequence of stimuli in which the attended feature (orientation, color or size) was manipulated and thus prioritized in memory. They found that attention to orientation (or color) significantly increased the attraction bias in a subsequent orientation (or color) report for an unrelated stimulus. Czoschke et al. [18] and Fischer et al. ([19], Exp.3) found that when participants encoded the motion direction of two random dot stimuli, SD occurred only when the stimulus on the preceding trial was cued for reporting. Later, Fischer et al. [20] confirmed the SD effect specifically toward the motion direction of the previously cued target but not the non-cued one. Additionally, they demonstrated that the direction of the cued target could be successfully reconstructed from MEG (Magnetoencephalography) data, whereas the non-cued target could not be reconstructed during the retro-cue phase. Hajonides et al. [28] obtained similar results with sequentially presented oriented patches, also using MEG. These results taken together suggest that prioritization in VWM is important in explaining the SD mechanism.

However, all studies comparing SD for cued and non-cued items used cues with 100% validity. That is, observers always reported or planned to report only the cued item. This might create a confound as the report can itself serve as a source of bias [19–21, 64, 65]. Thus, prioritized and not prioritized items are conflated with reported and non-reported ones. While Czoschke et al. ([18], Exp. 2) tried to circumvent this problem by omitting reports on some trials, [45] argued that a sensory representation undergoes a transformation in preparation for a response (resulting in a “decisional template”) that creates or amplifies SD even if the report is omitted. To avoid this confound, in our experiments, we aimed to compare the effects of reported cued and non-cued items (Exp. 1–3) in addition to the effects of non-reported cued and non-cued items (Exp. 2–3).

### Theoretical perspectives on the role of prioritization in SD

While SD has led to a large amount of empirical work, its theoretical understanding remains underdeveloped. Most of the proposed models remain at descriptive or mechanistic levels (in Marr’s, 1983, classification), limiting their capability to explain how prioritization can affect SD. However, two normative computational models, the Bayesian model [16, 55] and the Demixing Model [14], provide predictions for serial biases that depend on uncertainty or noise. Prioritization changes the reallocation of internal focus of attention or internal resources and is typically modeled as a change in the amount of internal noise (e.g., [7, 56]). Hence, these models are naturally suited to make predictions about prioritization’s effects on SD.

According to the Bayesian model, perception is a hypothesis about the external world shaped by sensory input [60]. However, as mentioned earlier, sensory information is often uncertain, prompting the brain to integrate priors and present inputs to enhance behavioral precision [55]. This strategy is adaptive in natural environments where the visual inputs are relatively stable across time. However, in the experimental conditions, when previous and current trials are unrelated, it leads to biases. Regarding prioritization, the Bayesian model proposes that the brain puts more weight on more reliable information. So, when the current stimulus is less uncertain compared to the previous one, SD magnitude decreases, and vice versa. In this way, observers make more accurate decisions by prioritizing information that is more certain, enhancing decision accuracy [55]. However, while standard Bayesian models explain the attractive biases in SD by integrating noisy sensory measurements of current and previous stimuli, they fail to account for repulsive biases effectively [24]. Extensions of Bayesian models — such as those incorporating efficient encoding and Bayesian decoding — have been proposed to capture both short-term attraction and longer-term repulsion patterns more accurately [24]. Nevertheless, these models fail to fully account for SD variability across different contexts, suggesting that the complexity of real-world perception introduces additional, sometimes contradictory, mechanisms.

In an attempt to provide a more parsimonious account, Chetverikov [14] proposed the Demixing Model, which suggests that SD, like other contextual biases, results from the observer’s attempt to separate neural signals related to different stimuli in the environment. In the case of SD, these are the signals related to the current and the previous items. Whether the bias is attractive or repulsive depends on factors such as item similarity, the level of sensory noise, and the observer’s assumptions about the environment. Prioritization is assumed to decrease sensory noise, which, according to the model, should in turn decrease the bias magnitude from previously encountered stimuli. However, Chetverikov [14] noted that in the case of biases between sequentially presented items, the pattern might be more complex. This is because the observer has already encoded and reported the previous item by the time they encounter the current one. Consequently, the initial noise level associated with the *previous* item might differ from the amount of noise in the representation of this previously seen item when the *current* item is presented. As an illustration, the perception of a noisy, low-contrast Gabor patch might be transformed into the representation of a single line, either real (e.g., a response bar) or imaginary, that might have a lower noise level.

We aimed to evaluate the predictions of the two models for SD using prioritization to manipulate the amount of noise in mnemonic representations. In addition to the studies on the role of attention in SD discussed above, the effect of noise and uncertainty on SD has been investigated in previous studies using stimuli manipulations with mixed results (here, we focus on attractive serial effects, while the repulsive effects have been studied elsewhere: e.g., [1–3, 13, 61]). Across several studies, the uncertainty in the current stimulus influenced the strength of SD, with stronger effects emerging when stimuli are less reliable, but no effect of previous stimulus uncertainty [11, 12, 16, 26], see also [25, 54]. Similarly, Little and Clifford [36] found that SD remained unaffected by either decisional or stimulus uncertainty of prior stimuli, including differences in stimulus or noise contrast. However, when looking at internal factors, both previous and current uncertainty might play a role. For example, van Bergen and Jehee [55] decoded uncertainty of visual representations from fMRI (Functional Magnetic Resonance Imaging) responses to show that a switch from low previous trial uncertainty to high current trial uncertainty leads to a particularly strong SD. In a similar vein, Markov et al. [41] found that manipulation of working memory load on both current and previous trials affects SD. It thus remains possible that different types of manipulations — particularly those targeting more internal noise — could reveal an effect of previous stimulus uncertainty on SD. All in all, while there is evidence that the precision of internal representations affects SD, the attempts to empirically manipulate uncertainty in current and previous trials focused mostly on external factors.

This difference suggests that attentional focus or uncertainty alone does not fully account for the observed effects, and additional factors influence whether the items in memory are protected from interference. However, the lack of effect observed in these studies does not mean that we should exclude previous stimulus uncertainty from the SD framework [26]. It remains possible that different types of manipulations — particularly those targeting more internal noise — could reveal an effect of previous stimulus uncertainty on SD. All in all, while there is evidence that the precision of internal representations affects SD, the attempts to empirically manipulate uncertainty in current and previous trials focused mostly on external factors (e.g., contrast or spatial frequency) with mixed success.

In sum, this study tests the Bayesian observer and the Demixing Model predictions about serial dependence using VWM or attentional prioritization to modulate internal noise in observers’ representations. In addition, in Experiments 2 and 3, we cover the gap in the previous literature by separating the attentional prioritization from the presence or absence of report. We will next present the computational models that formalize our hypotheses regarding the influence of prioritization on SD. First, we will recreate the Bayesian observer model, which explains SD as a rational strategy under uncertainty. Then, we will introduce the Demixing Model [14] predictions for SD.

## Computational models

### Bayesian observer

#### Model

To illustrate the Bayesian model predictions, we simulated the behavior of a Bayesian observer as described by van Bergen and Jehee [55].

The model starts with the assumption that in each trial, the observer obtains a sensory measurement (*x*) of stimuli (*s*) corrupted by noise:

1 $$\begin{array}{c}p\left(x\vert s\right)=f_{WN}\left(x;s,\sigma^2\right)\end{array}$$

Here, we use a wrapped normal noise distribution to account for circularity in the orientation space, with its variance ${\sigma }^{2}$ reflecting the amount of noise.

The observer also assumes that the stimuli on consecutive trials are related to each other following the statistics of the natural environment:

2 $$\begin{array}{c}p\left(s_t;s_{t-1}\right)=p_\text{same}C\left(s_t;s_{t-1},\sigma_s,\gamma\right)+\left(1-p_\text{same}\right)U\left(\text{0,2}\pi\right)\end{array}$$

where ${s}_{t}$ is the stimulus on the current trial, ${s}_{t-1}$ is the stimulus on the previous trial, ${p}_{\text{same}}$ is the probability that there was no abrupt change in the environment, ${\sigma }_{s}$ is the standard deviation of the peak of the function $C$, and $U(\text{0,2}\pi )$ is the circular uniform distribution. The function *C* describes the probability of stimulus changing between the trials in the absence of abrupt changes:

3 $$\begin{array}{c}C\left(s_t;s_{t-1},\sigma_s,\gamma\right)=\frac1Z\text{exp}\left(-\frac1{2\sigma_s^2}\vert\text{angle}\left(s_t,s_{t-1}\right)\vert^\gamma\right)\end{array}$$

where *Z* is the normalization constant, $\text{angle}({s}_{t},{s}_{t-1})$ is the angular difference between the stimuli, and $\gamma$ determines the steepness of the function.

The observer then inverts this generative model (Eqs. 1 and 2) to infer the probability of different stimuli in each trial using Bayes’ rule. In other words, the observer combines information about the current and the previous stimuli based on the measurements obtained in the two trials and the assumed relationship between the stimuli:

4 $$\begin{array}{c}p\left(s_t\vert x\right)\propto p\left(x\vert s_t\right)p\left(s_t\vert s_{t-1}\right)\end{array}$$

This final distribution represents the observer’s belief about the orientation of the current stimulus based on the sensory observations about the current and the previous stimuli. The observer then uses the mean of the posterior distribution as a response.

#### Simulations

To simulate the observer behavior, we first randomly picked the stimuli for 10^6^ trials. By adding the noise to each stimulus (with the high or low level of noise assigned randomly), we created a vector of sensory observations across trials. For each sensory observation, we then computed the likelihood using a wrapped normal distribution function, with the mean based on the observation and the variability determined by the trial noise. In alignment with our first experiment, the amount of noise ($\sigma$) is modulated by prioritization: when a target was cued, it represented a low noise condition, and when a target was non-cued, it represented a high noise condition (6, 9 and 12°, respectively, converted to radians). Only three levels of noise were used because the predictions of the Bayesian observer model are relatively straightforward and have been described before (e.g., [16, 55]). To calculate the prior distribution, we used a uniform prior for the first observation, and for subsequent observations, we used the previous posterior convolved with a transition kernel (Eq. 2). For our simulations, we used the values of ${\sigma }_{s}=$ 16.9, $\gamma =$ 2.6, and ${p}_{\text{same}}=$ 0.64 based on van Bergen and Jehee [55]. We evaluated errors in the observer’s responses by comparing these estimates to the actual orientation of the stimulus for each observation and computed the bias by multiplying errors by the sign of the distance to the previous target on each trial.

**Fig. 1:**
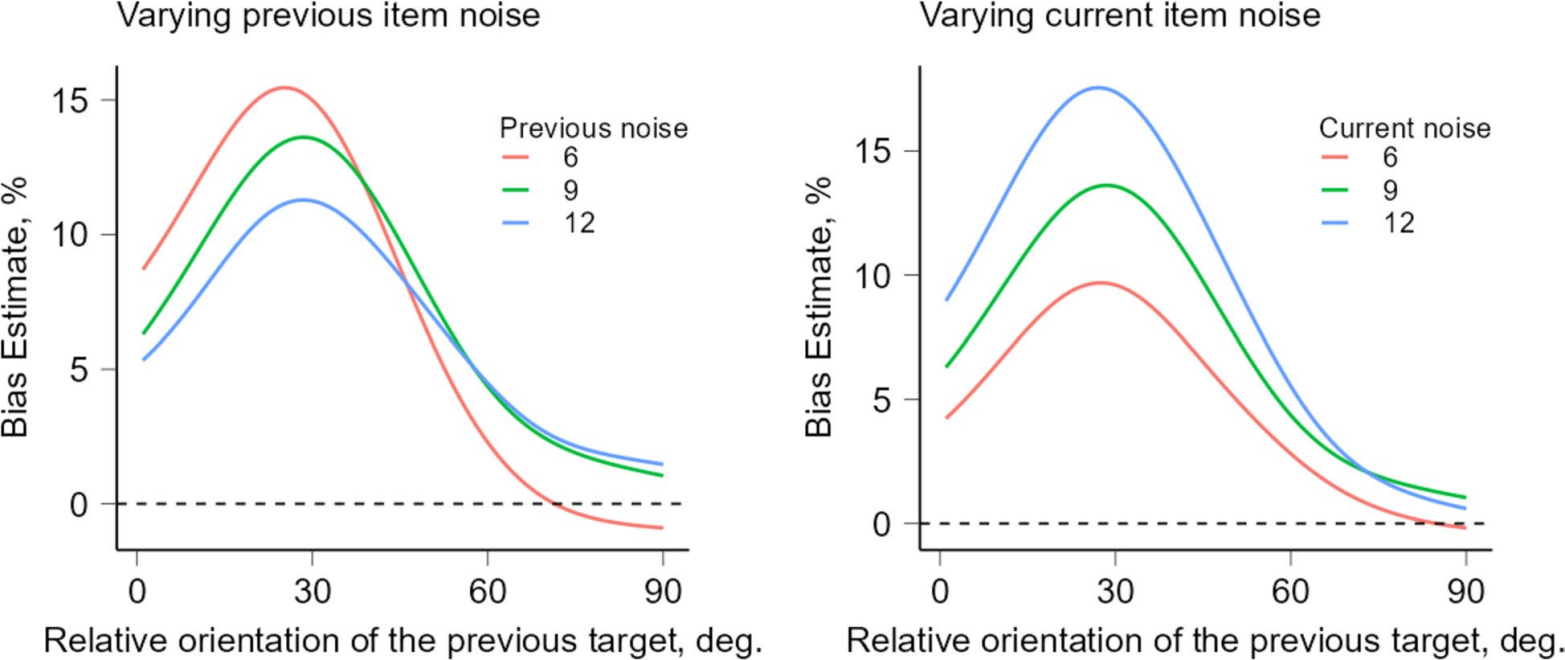
A Bayesian observer model for serial dependence effect with two levels ofcueing for the current and the previous target*. *The left plot shows the effect of varying previous stimulus noise while keeping the current stimulus noise fixed at 9°. The right plot shows the effect of varying current stimulus noise while keeping the previous stimulus noise fixed at 9°. Biases in orientation estimates (in degrees) for responses as a function of dissimilarity between current and previous stimuli. Positive values indicate an attractive bias

Finally, we followed the procedure used for the actual data (see Methods) to estimate SD across trials (see Fig. 1). We plotted bias against orientation differences between consecutive stimuli to illustrate how SD was affected by the noise level of the previous and current target. The model predicted an attractive bias, showing a linear increase in SD as the previous trial’s noise decreased, and a weaker SD when the current trial’s noise decreased. This is consistent with the idea that the relative weight of past information increases when the current input is more uncertain [55]. The model further demonstrates that when previous information has a higher relative weight (such as when it is prioritized), it increases the amplitude of SD.

### The Demixing Model (DM)

#### Model

We derived DM predictions for serial dependence following the approach described by Chetverikov [14]. DM assumes that in each trial, the observer obtains multiple sensory measurements **x**. Each sensory measurement comes from one of two sources (or components), with probabilities ${\pi }_{1}$ and ${\pi }_{2}=1-{\pi }_{1}$. One component here represents a recent stimulus that the participant has to remember, while the other component represents a previous stimulus that is no longer relevant but may still influence perception.

These measurements capture information across two key perceptual dimensions. The first dimension represents the reported feature, which is, for our purposes, orientation. Due to its circularity, we modeled orientation using a wrapped normal distribution. The second dimension encompasses all other features that are not reported but allow observers to determine which item to report — for example, its color, spatial location, or other distinguishing characteristics. In our case, the most prominent feature here is time, as observers must report the last item they saw rather than one from a previous trial. Unlike orientation, this dimension is not circular and its samples are normally distributed. In sum, both components are characterized as a bivariate wrapped normal — normal distribution with a diagonal covariance matrix (i.e., no correlation between the components).

Each of these two components has its own characteristics: a mean value (${\mu }_{1,j}$, ${\mu }_{2,j}$) and standard deviation (${\sigma }_{1,j}$, ${\sigma }_{2,j}$) for both the orientation and temporal dimensions, where $j\in \{1,2\}$ indicates the component. The mean corresponds to the true stimuli parameters (e.g., an orientation of 45° and a specific time point), while the standard deviation quantifies the noise in neural processing.

When modeling the probability of observing a particular measurement ${x}_{i}=\left({x}_{i,1},{x}_{i,2}\right)$, the probabilities from both components are combined﻿:

5 $$\begin{array}{c}p\left(x_i\vert\theta\right)=\sum\limits_{j=1}^2\pi_j\left[f_{WN}\left(x_{{i,}{1}};\mu_{1,j},\sigma_{1,j}^2\right)\cdot f_N\left(x_{i,2};\mu_{2,j},\sigma_{2,j}^2\right)\right]\end{array}$$

where $\theta =\{\pi ,\mu ,\sigma \}$ is the set of all parameters, ${f}_{WN}$ is the wrapped normal distribution (for orientation), and ${f}_{N}$ is the standard normal distribution (for the temporal dimension). The equation assumes independence between the orientation and temporal dimensions within each component, allowing the joint probability to be expressed as the product of the individual dimension probabilities.

Based on these sensory measurements, the observer determines the most likely values for the means and standard deviations of the two components through maximum likelihood estimation:

6 $$\begin{array}{c}\theta= \text {argmax}L\left(\theta;\mathbf x\right)\end{array}$$

The behavioral response is then determined by selecting the estimated orientation mean (${\widehat{\mu }}_{1,j}$) from the component with a higher value on the temporal dimension (larger ${\widehat{\mu }}_{2,j}$). This selection process models how observers identify the most recent or temporally relevant stimulus when making their response.

#### Simulations

In the simulations, noise levels ($\sigma$) were manipulated to represent different prioritization conditions. When a stimulus is cued as important (high priority), it is modeled with lower noise, reflecting more precise encoding. When a stimulus is not cued (low priority), it is modeled with higher noise, reflecting less precise encoding. At the same time, we assumed that the previously shown item always has higher noise levels than the current one, due to memory decay.

In the case of the Bayesian model, predictions related to noise have been previously described in the literature and are relatively straightforward, making the number of noise levels a less critical factor. Therefore, we used only three levels of noise for both current and previous noise. In contrast, predictions from the Demixing model are more complex and have not yet been described. Initial simulations suggested that the model’s predictions do not vary in a straightforward or monotonic way with noise. Because of this, we could not assume in advance how it would respond to different levels of uncertainty. To explore these predictions in more detail, we introduced additional levels of noise for the current item, allowing for a more comprehensive examination of the model’s behavior.

Based on the preliminary exploration of the parameter space, different levels of noise in orientation perception were examined by testing five different standard deviation values (${\sigma }_{1,1}$ ranging from 12 to 28° in 4° steps) for the first component (representing the stimulus in the current trial) and three different levels (${\sigma }_{1,2}\in \{40^\circ , 60^\circ , 80^\circ \}$) for the second component (representing the previous stimulus). These values were selected so that behavioral variability of the model lies in the same range as the behavioral variability of the real observers and the overall direction of biases for the low-noise item remains positive, corresponding to the typical pattern of SD effects. The temporal standard deviation ${\sigma }_{2}$ was assumed to be equal for both components and fixed (${\sigma }_{2}={\sigma }_{\text{2,1}}={\sigma }_{\text{2,2}}=20$) and the discriminability in the temporal dimension was fixed as well (${d}{\prime}_{\text{temp}}=\frac{{\mu }_{2,1}-{\mu }_{2,2}}{{\sigma }_{2}}=1$). The number of observations was fixed at $N=100$ measurements in our simulations and the probabilities of signals being caused by each component were assumed equal (${\pi }_{1}={\pi }_{2}=0.5$). These parameters represent the “average” case considered by Chetverikov [14] and do not cover the full space of potential model behavior. Our explorative analysis of other parameters suggests that they do not affect the direction of the previous item’s noise effect, while the current noise effect can become positive-only or negative-only (with respect to the changes in bias) in addition to the inverted U-shaped pattern described below. Finally, the orientation difference between the two components was systematically varied across 120 steps from 0 to 90°, representing conditions where the two orientations range from identical (0° difference) to maximally different (90° difference).

For each combination of parameters, we simulated 10,000 trials. For each trial, sensory measurements were generated according to the true model, then the Expectation–Maximization algorithm was employed to obtain the maximum likelihood estimate of the model parameters. To ensure convergence to the global optimum, 50 different random initializations were used for each simulation.

**Fig. 2:**
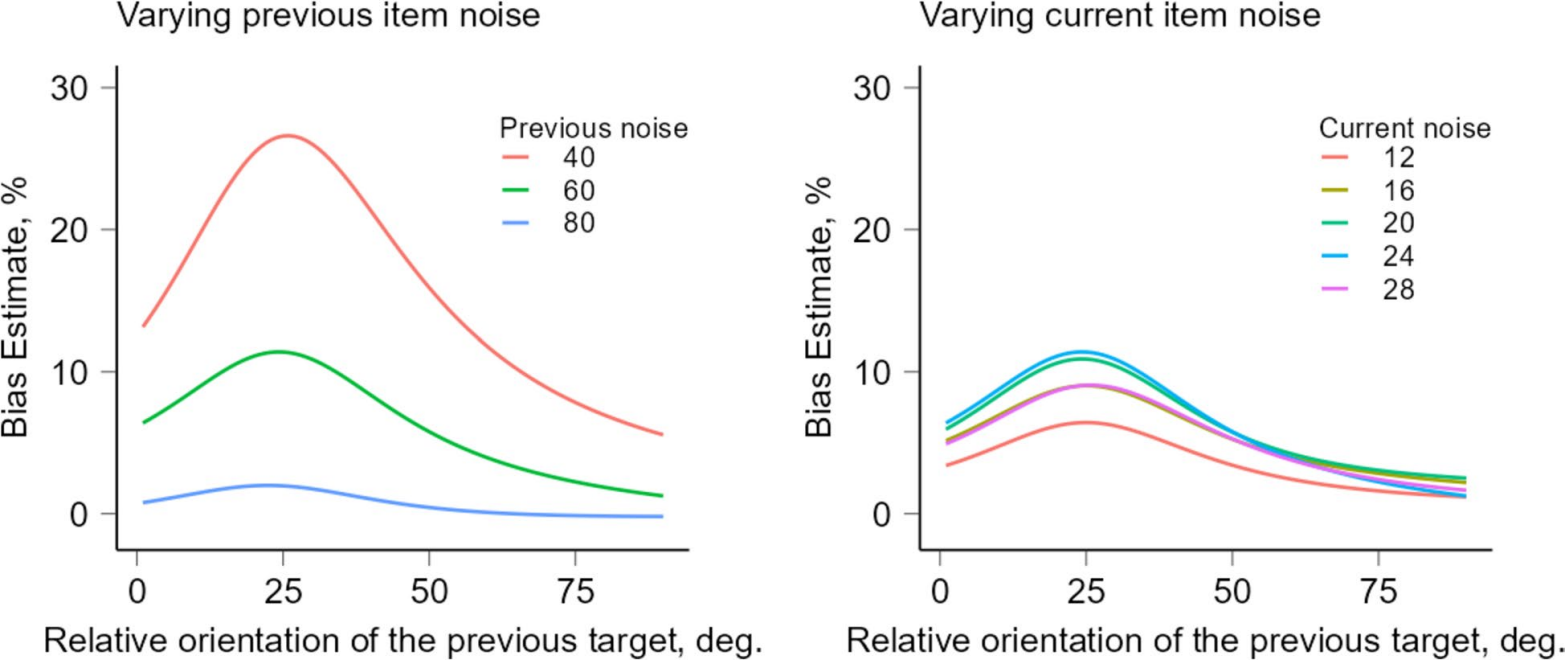
A Demixing Model for serial dependence effect with varying levels of noise for the current and the previous stimuli*.* The left plot shows the effect of varying previous stimulus noise while keeping the current stimulus noise fixed at 24°. The right plot shows the effect of varying current stimulus noise while keeping the previous stimulus noise fixed at 60°. Biases in orientation estimates (in degrees) for responses as a function of dissimilarity between current and previous stimuli. Positive values indicate an attractive bias

Finally, to generate predictions, we estimated the SD across trials using the same procedure applied to the actual data. Figure 2 shows the effects of the previous noise with current stimulus noise ($\sigma_{1,1}$) fixed at 24° along with the effect of the previous stimulus noise (${\sigma }_{1,2}$) fixed at 60°. The simulated results show a linear increase in SD as the noise of the previous item decreases — a prediction similar to the one of the Bayesian model. However, varying the noise of the current item produces a non-linear, inverted U-shaped dependence. The strongest bias in Fig. 2 (right panel) is observed at the intermediate level of noise for the current item ($\sigma_{1,1}=24^\circ$). This is because as the noise in the current item becomes more similar to the previous one, the bias for the current item generally shifts from attraction to repulsion that is expected when the noise levels are equal (see [14]).

### The current study

We explored how prioritization in memory influences SD in three experiments. In Experiment 1, we manipulated prioritization in a standard delayed report task with a single-oriented Gabor stimulus in each trial. To this end, in some trials, we presented a cue before the stimulus (“precue”), indicating to participants that they would need to additionally report the stimulus the second time after the next trial. We hypothesized that it would increase the priority of this stimulus during the retention interval, creating stronger serial dependence as predicted by both the Bayesian Observer model and the Demixing Model. The Bayesian model additionally predicts that prioritization should decrease the amount of noise in the current trial and make the prioritized stimulus less susceptible to biases. In Experiments 2 and 3, we examined the effect of simple attentional prioritization through pre- or post-cueing of one of two stimuli with a cue probabilistically indicating the item participants would have to report. Similarly, we expected that cued items should be less affected by SD from previous trials (as predicted by the Bayesian Observer model) and, in turn, induce stronger SD in the following trials (as predicted by both the Bayesian Observer and the Demixing Model).

Previewing the main results, we found that in Experiment 1 the additional requirement to hold information in memory for a longer period of time increased the strength of SD created by the item held in memory. In contrast, in Experiments 2 and 3, we found no significant differences between congruent and incongruent conditions, indicating that manipulating uncertainty through pre- or post-cueing for one of two simultaneous stimuli did not affect SD. This is despite the clear evidence of prioritization in the form of reduced error variability for cued items in all three studies. Our findings suggest that active memory maintenance can amplify perceptual biases beyond mere attentional prioritization effects. This opens pathways for refining models of SD in perceptual judgment tasks.

## Results

### Experiment 1

**Fig. 3:**
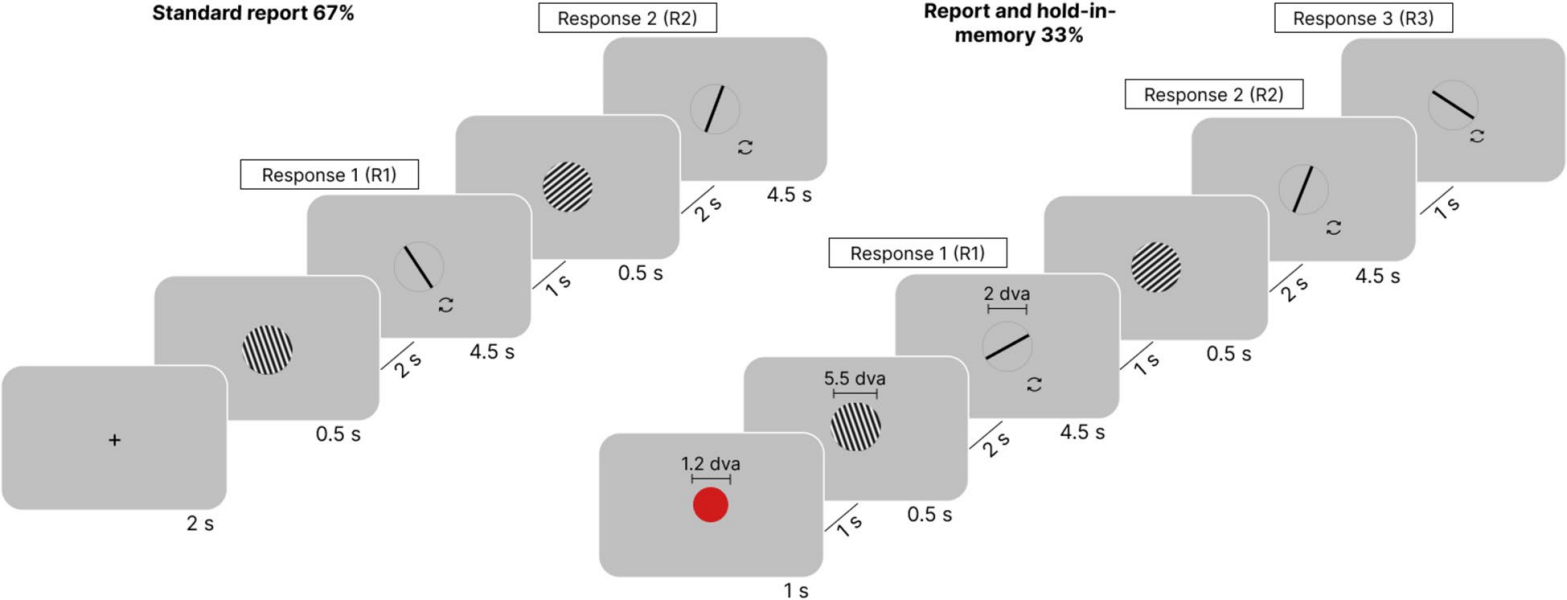
Design of Experiment 1*.* In the standard procedure (67% of trials), participants viewed a Gabor patch and were required to report its orientation immediately after the presentation. In the report-and-hold-in-memory condition (33% of trials), participants first received a cue before the initial Gabor patch and reported the orientation of the first Gabor twice: once immediately after its presentation (Response 1) and again (Response 3) after reporting the orientation of the second Gabor (Response 2). The stimuli depicted in the figure are not drawn to scale. The fixation cross was also shown during the intervals between stimuli and reports (not drawn for conciseness). The circular arrows shown near the response bar were not part of the actual experiment

In Experiment 1, participants viewed pairs of Gabor patches (“streaks”) and performed orientation adjustment tasks. In two randomly interleaved conditions, they either reported each stimulus only once immediately after the presentation of the stimulus (standard report) or, if a red circle appeared before the first patch, they were instructed to memorize its orientation and report it twice: once immediately after the presentation and again after response on the second stimulus (the report-and-hold-in-memory condition; see Fig. 3 and Methods for details).

#### Overall performance

**Fig. 4:**
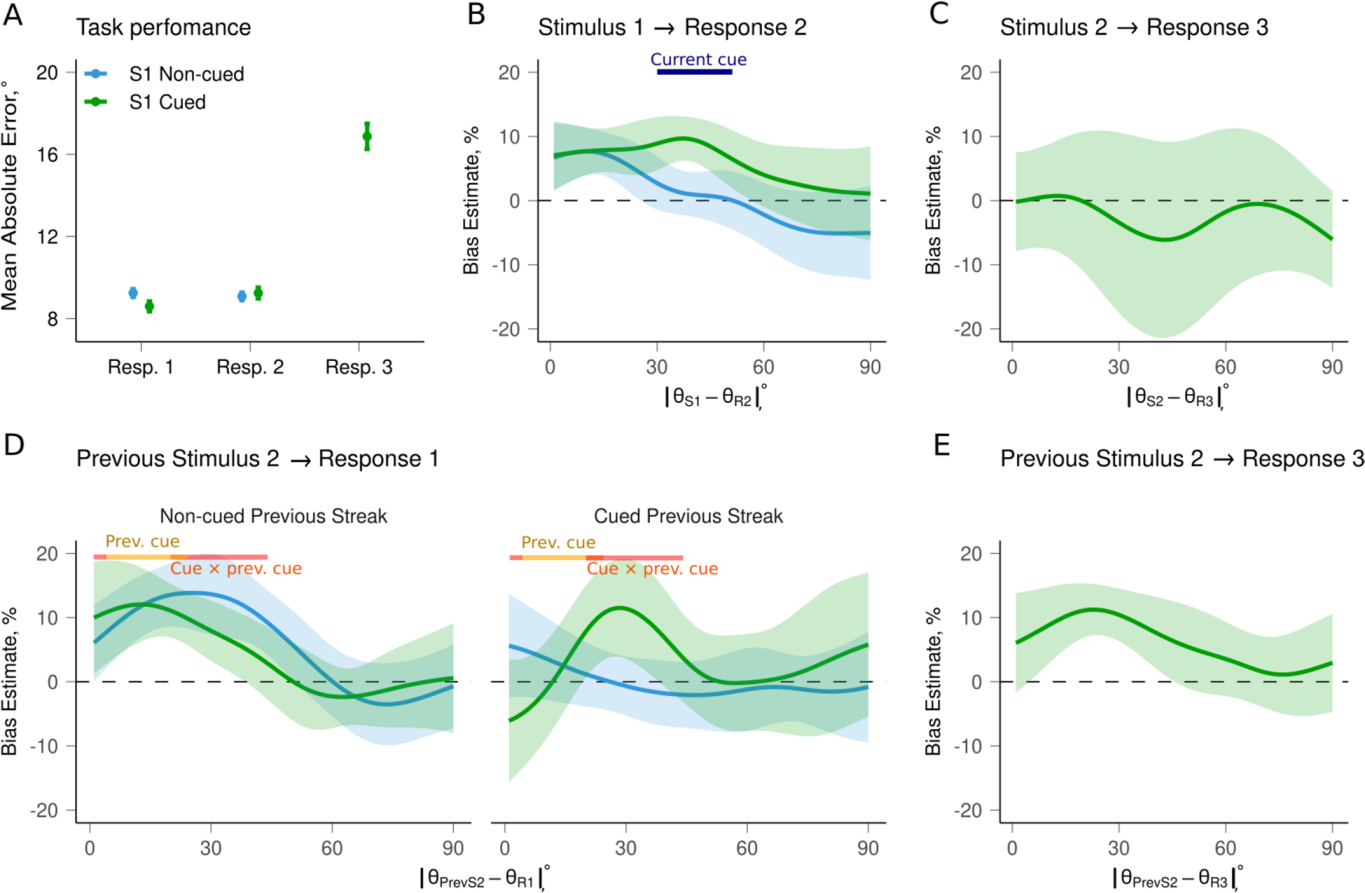
Task Performance and Serial Dependence Plots (*N* = 18 participants, *N*_trials_ = 648). **A** Mean absolute error in orientation estimates in each condition. Bars show 95% confidence intervals (CI). **B–E** Biases in orientation estimates for Responses 1–3 as a function of dissimilarity (angular difference) between the current and previous stimuli. Positive values correspond to attractive bias (serial dependence). Shaded regions show 95% CI. The horizontal segments above the lines indicate the dissimilarity range where the effects of conditions (labeled near the lines) were significant. The term “previous” refers to the previous streak. =

We first tested the effectiveness of the pre-cue manipulation, which involved holding a stimulus in memory for cued stimuli (see Fig. 4A). As expected, participants had smaller errors for the immediate report (Response 1 to Stimulus 1) of cued compared to non-cued stimuli (*M* = 8.59, *SD* = 7.82, vs. *M* = 9.24, SD = 8.89, *t*(35) = 4.03,* p* < 0.001). In contrast, Response 2 to Stimulus 2 was slightly negatively affected when observers had to hold another item in memory, with larger errors for cued compared to non-cued streaks (*M* = 9.24, SD = 8.61, vs. *M* = 9.08, *SD* = 8.64, *t*(35) = − 0.93,* p* < 0.001). The delayed Response 3 to Stimulus 1 resulted in larger errors compared to Response 1 and Response 2 (*M* = 16.88, *SD* = 19.73; *p* < 0.001 for both comparisons).

#### Data preprocessing

Participants’ responses were preprocessed to remove idiosyncratic orientation-dependent bias (i.e., individual variations in perception based on stimulus orientation) from reports and to identify and remove outliers for each participant using the *remove_cardinal_biases* function in the *circhelp* package in R [15].

We estimated biases relative to previously seen items by calculating the asymmetry in response probability density. This measure indicates that it was much more likely that participants made an error in the direction of the previous item than in the direction away from it for each angular distance between items. The *density_asymmetry* (*circhelp* package in R; [15]) function was utilized to generate a smoothed estimate of the asymmetry in error probability as a function of dissimilarity (angular difference) between the current and the previous stimuli. When estimating probabilities for each dissimilarity step, we considered not only trials with the same difference but also all trials to capture a more continuous and nuanced probability distribution of errors. However, trials that were closer in difference to the current one were given higher weight in the asymmetry estimate. Analyzing the asymmetry in probability allowed us to identify clearer patterns in participants’ responses than relying solely on mean bias values.

The complete preprocessing and analysis code is available on the Open Science Framework (OSF) and can be accessed via the following link: https://osf.io/wunf8/?view_only=3ddc35f3c3ac4ffc8947a56200ffe4c5

#### Serial dependence effect within a streak

We first looked at the average SD magnitude (i.e., the asymmetry in response probability density, see Data Preprocessing) for Response 2. As mentioned earlier, we define pairs of Gabor patches as “streaks.” Accordingly, here we examine SD within a single streak. The results showed that across all trials, Response 2 was attracted by Stimulus 1 only when the latter was cued (*M* = 0.06, *SD* = 0.14, *t*(17) = 2.69,* p* = 0.011) but not when it was non-cued (*M* = 0.01, *SD* = 0.09, *t*(17) = 0.78, *p* = 0.442), based on mean bias, calculated as mean signed errors with their signs reversed.

We then analyzed the SD as a function of the angular difference between the stimuli to determine more specific regions where bias persists. Because SD effects are often strongest at specific angular separations rather than across the entire range, we examined each one-degree step from 0 to 90° (absolute difference, due to circular symmetry). For each angular difference step, we computed separate one-way *t*-tests for the cued and non-cued conditions to determine whether bias at that step was significantly different from zero. To ensure stable estimates despite the small number of trials at any exact degree, we used a smoothed kernel approach in which all trials contributed to each step but were weighted by proximity in angular difference. This allowed us to identify contiguous regions showing consistent attractive or repulsive bias within each condition. This more detailed analysis by angular distance revealed a significant attractive bias in cued streaks at distances of 5–50° (*t*(17) > = 2.07, *p* < 0.05), as well as in non-cued streaks at distances of 1–25° (*t*(17) > = 2.13, *p* < 0.05), along with a repulsive bias at distances of 70–80° (*t*(17) > = − 2.05, *p* < 0.05). This combination of opposing biases explains the null results with the non-cued streaks when the average bias is considered. In essence, in the report-and-hold-in-memory condition, the report (and hence the representation) of stimulus orientation in a given streak is biased (attractively) by the orientation of the previous stimulus when past and present stimuli are moderately similar (the strongest effect occurs at around a 35-degree difference between the two stimuli). We then applied a repeated-measures ANOVA to assess differences between the cued and non-cued conditions at each one-degree step. Again, rather than interpreting isolated points, we focused on clusters of consecutive degrees with consistent effects. Analysis showed significant differences between cued and non-cued streaks when the differences between the two stimuli were in the 30–51° range (*F*(1,17) > = 4.68, *p* < 0.05) (see Fig. 4B). Participants exhibited stronger SD, extending across larger angular differences, when required to retain previous stimuli in memory.

The results did not show any dependence between Stimulus 2 and Response 3; the representation of Stimulus 1 held in memory and reported the second time (*M* = −0.03, *SD* = 0.22, *t*(17) = −0.71, *p* = 0.483; Fig. 4C). This suggests that a stimulus with a previously given response might be less vulnerable to external visual interference.

#### Serial dependence effect across different streaks

We then analyzed the interactions across different streaks, where the previous streak refers to the previous pair of stimuli. From the participant’s perspective, when both consecutive streaks are uncued, there is no meaningful difference between one streak and the next. However, we distinguish between streaks for analytical purposes because Stimulus 1 within each streak can be cued, whereas Stimulus 2 is always uncued. Thus, when a streak begins with a cued S1, this cue can modulate subsequent responses in ways that do not occur in uncued streaks.

In general, Response 1 to Stimulus 1 of the current streak was attracted towards Stimulus 2 of the previous streak, but SD was significant only for non-cued ones based on the mean bias (cued: *M* = 0.03, *SD* = 0.10, *t*(17) = 1.90, *p* = 0.065; vs. non-cued: *M* = 0.04, *SD* = 0.09, *t*(17) = 2.30, *p* = 0.027; Fig. 4D). However, when considering the dissimilarity between the stimuli, the influence of cueing in the previous streak was evident with attractions of Response 1 of the current streak in the 5–24 degree range (*F*(1,17) > = 4.92, *p* < 0.05), whereas the interaction between previous and current streak cueing was observed in the 1–4 degree and 20–44 degree ranges (*F*(1,17) > = 4.52, *p* < 0.05). Figure 4D shows a weaker bias when the current target was cued but there was no cue in the previous streak. This supports the idea that prioritization improves the resilience of the representation of the current target. However, when the previous streak was cued, the bias from Stimulus 2 was absent for non-cued current Stimulus 1, likely due to the presence of additional interfering representations (Stimulus 1) and responses (Response 3) from the previous streak, which may interfere with the representation of Stimulus 2. Interestingly, SD was observed only for cued current streaks and not for non-cued ones, which was unexpected. It was anticipated that increased prioritization of the current trial would reduce the bias, yet this result suggests otherwise in the case of additional interventions. Overall, this implies that bias from the previous streak can persist, even when current stimuli are prioritized.

SD was observed not only in the immediate response to Stimulus 1 (that is, Response 1) but also in the delayed response to Stimulus 1 (that is, Response 3) (*M* = 0.06, *SD* = 0.10, *t*(17) = 3.48, *p* = 0.001) (Fig. 4E). In other words, Stimulus 2 from the previous streak could influence the representation of subsequent Stimulus 1, and this influence persisted longer (up to Response 3).

### Experiment 2

**Fig. 5:**
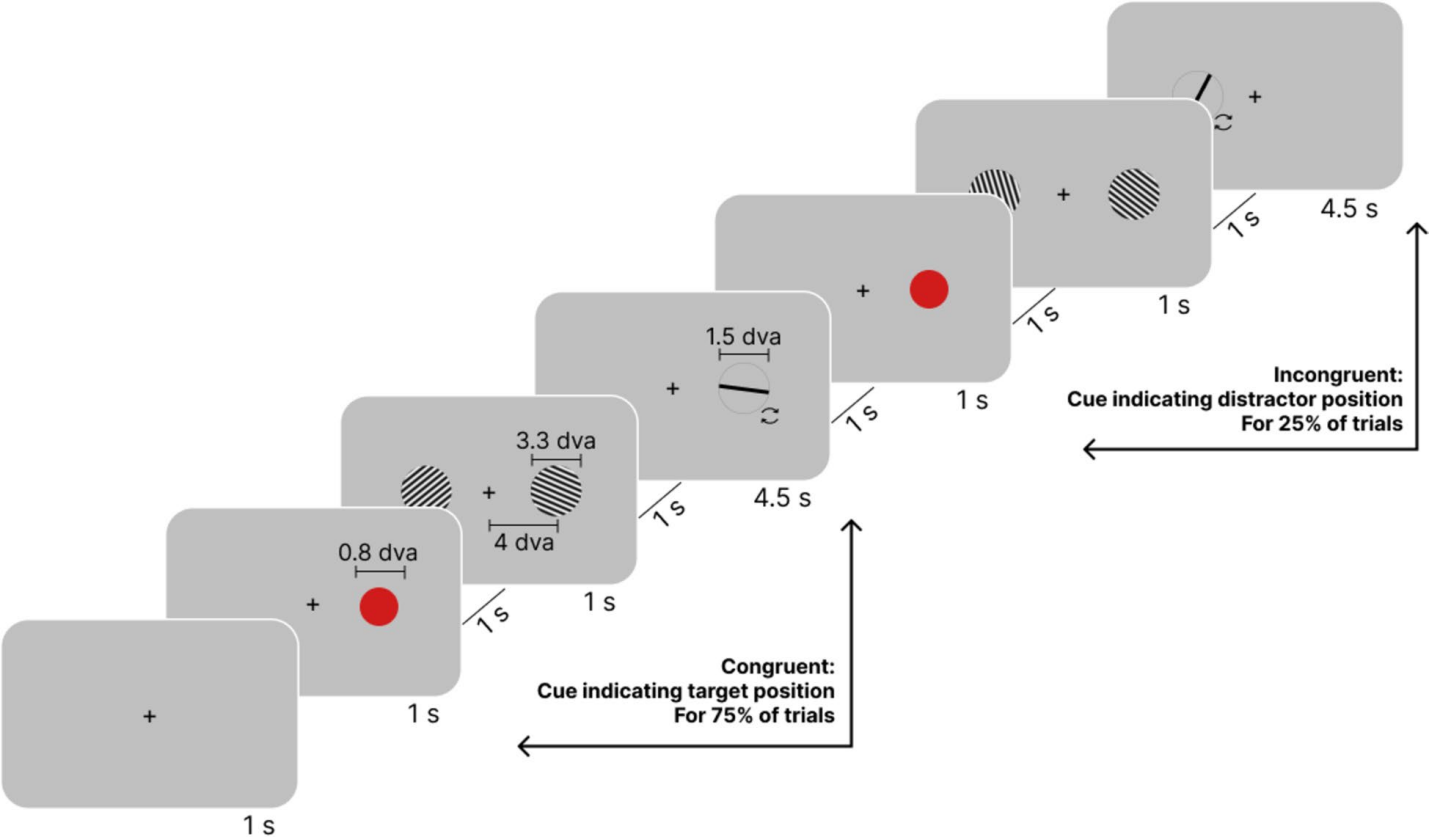
Design of Experiment 2*.* Participants reported the orientation of a Gabor patch at the location matching the adjustment bar location. This Gabor patch was either cued (congruent condition, 75% of trials) or non-cued (incongruent condition, 25% of trials). The stimuli are not drawn to scale. The fixation cross was also shown during the intervals between stimuli and reports (not drawn here). The circular arrows shown near the response bar were not part of the actual experiment

Experiment 2 tested whether the effects of prioritization on the magnitude of SD were due to increased attention to the cued stimulus. We presented participants with pairs of Gabor patches and asked them to report the orientation of one of the stimuli based on a pre-cue indicating the target side. In 75% of the trials (congruent condition), participants reported the orientation of the cued patch; in 25% of the trials (incongruent condition), they reported the non-cued patch (see Fig. 5 and Methods for details).

#### Data preprocessing

The analysis for Experiment 2 followed the same steps as in Experiment 1. For this experiment, we created one variable with three levels for previous and current congruence: incongruent-congruent, congruent-incongruent, and congruent-congruent. Incongruent-incongruent pairs of trials were not analyzed as they constituted a small portion of trials (around 45 for each participant).

#### Overall performance

**Fig. 6:**
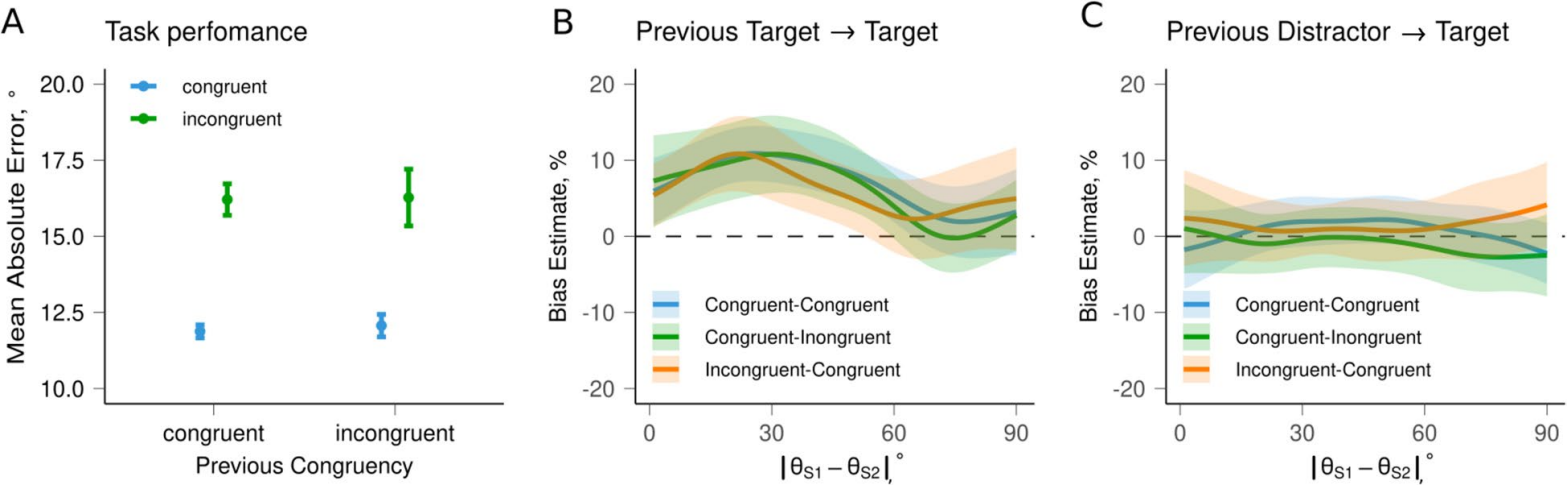
Results of Experiment 2 (*N* = 36 participants, *N*_trials_ = 720). **A** Overall performance. Mean absolute error (bars) with 95% confidence intervals (error bars) for each condition. **B**, **C** Serial dependence as a function of the angular difference between consecutive targets (**B**) or between the previous distractor and the current target (**C**). Lines show the mean bias; shaded regions show 95% confidence intervals

A comparison of mean errors across conditions confirmed the effectiveness of the congruence manipulation between the pre-cue and response location, with better performance in the congruent condition during the current trial (see Fig. 6A). Specifically, participants exhibited significantly smaller response errors in the congruent condition (*M* = 11.92, *SD* = 13.18) compared to the incongruent condition (*M* = 16.22, *SD* = 18.58), as indicated by the effect of current congruency (*F*(1,35) = 10.82, *p* = 0.002). However, no significant differences were found for previous congruency (*F*(1,35) = 0.19,* p* = 0.669) or their interaction (*F*(1,35) = 0.03, *p* = 0.871).

#### Serial dependence effect

The analysis revealed a classic SD effect for two sequential targets across conditions, with participants consistently reporting the current target as being attracted to the previous target (*M* = 0.07, *SD* = 0.12, *t*(35) = 8.21, *p* < 0.001). We then analyzed SD in reports based on the congruency of the current and previous trials, calculating the difference between conditions for each 1-degree step of similarity (angular difference) between the current and previous targets. A one-way ANOVA showed no significant differences in the strength of SD between congruent and incongruent conditions, regardless of the dissimilarity between the current and previous targets (see Fig. 6B). Similarly, the ANOVA on the mean values showed no significant effect of condition (*F*(2, 70) = 0.09, *p* = 0.881). These results suggest that prioritization by means of increased attention to the item before encoding did not influence the magnitude of SD. To ensure the robustness of our results, we performed a Bayesian ANOVA on the average bias computed for each participant across dissimilarity levels. The Bayes factors (*BF*_10_ = 0.09 ± 0.89%) provided strong evidence in favor of the baseline model (bias predicted by the random effect of participant only) over models including congruency and previous congruency, suggesting that these factors did not significantly impact bias, with the null hypothesis being more likely than the alternative.

In an exploratory analysis, we also estimated the location of the maximum bias point (a “peak”) for SD curves in the orientation dissimilarity space for each participant in each condition. A Bayesian ANOVA indicated that the peak locations were not affected by the condition (*BF*_10_ = 0.15 ± 0.88%).

Additionally, we examined whether there is a difference in SD from non-reported items (distractors) compared to reported ones, and whether a non-reported cued item differs from a non-reported non-cued item. The analysis did not reveal a significant SD effect from the previous distractor to the current target stimulus across conditions (*M* = 0.003, *SD* = 0.10, *t*(35) = 0.47, *p* = 0.639), and we found no significant differences between the conditions based on the difference between conditions for each 1-degree step of similarity (see Fig. 6C). The ANOVA on the mean values also revealed no significant effect of condition (*F*(2, 70) = 1.78, *p* = 0.179). For the differences in SD from non-reported items compared to reported ones, a paired-samples *t*-test showed that SD was significantly stronger for the previous target than for the previous distractor (*t*(35) = 5.95, *p* < 0.001). This suggests that probabilistic cueing does not play a substantial role when the presence or absence of reporting is taken into account.

### Experiment 3

**Fig. 7:**
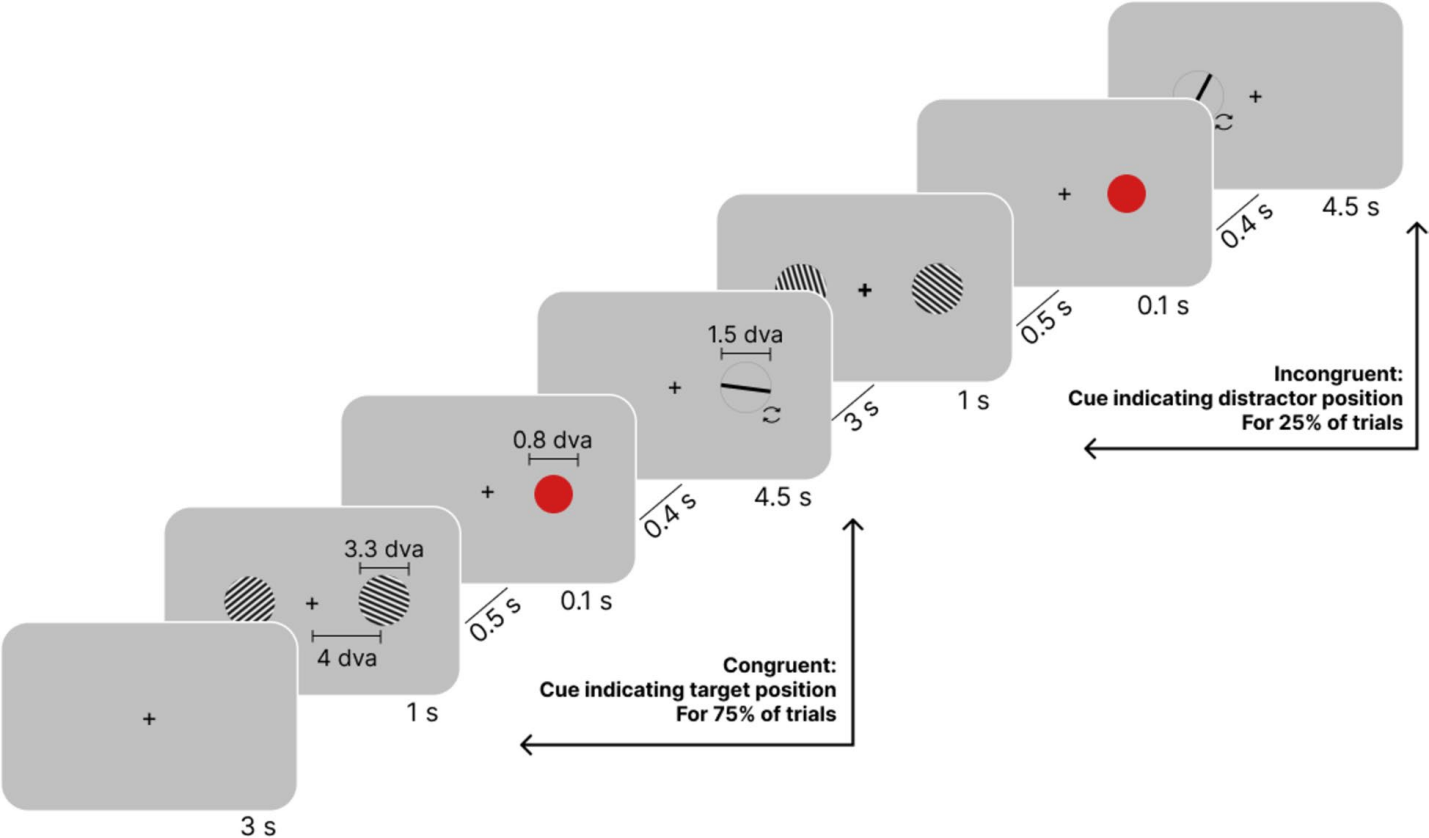
Design of Experiment 3*. *Participants reported the orientation of a Gabor patch at the location matching the adjustment bar location. This Gabor patch was either cued (congruent condition, 75% of trials) or non-cued (incongruent, 25% of trials). The stimuli depicted in the figure are not drawn to scale. The fixation cross was also shown during the intervals between stimuli and reports (not drawn for conciseness). The circular arrows shown near the response bar were not part of the actual experiment

Experiment 3 examined if and how prioritization by means of increased attention to encoded items in the early phase of VWM maintenance influences the magnitude of SD under post-cueing conditions. The design of Experiment 3 was identical to that of Experiment 2, except that the target side was indicated using a post-cue rather than a pre-cue. Participants viewed pairs of Gabor patches and reported the orientation of one stimulus based on this cue. In 75% of trials (congruent condition), the cue matched the target patch; in 25% (incongruent condition), it pointed to the non-target (see Fig. 7 and Methods for details).

#### Data preprocessing

The data analysis for Experiment 3 was identical to Experiment 2.

#### Overall performance

**Fig. 8:**
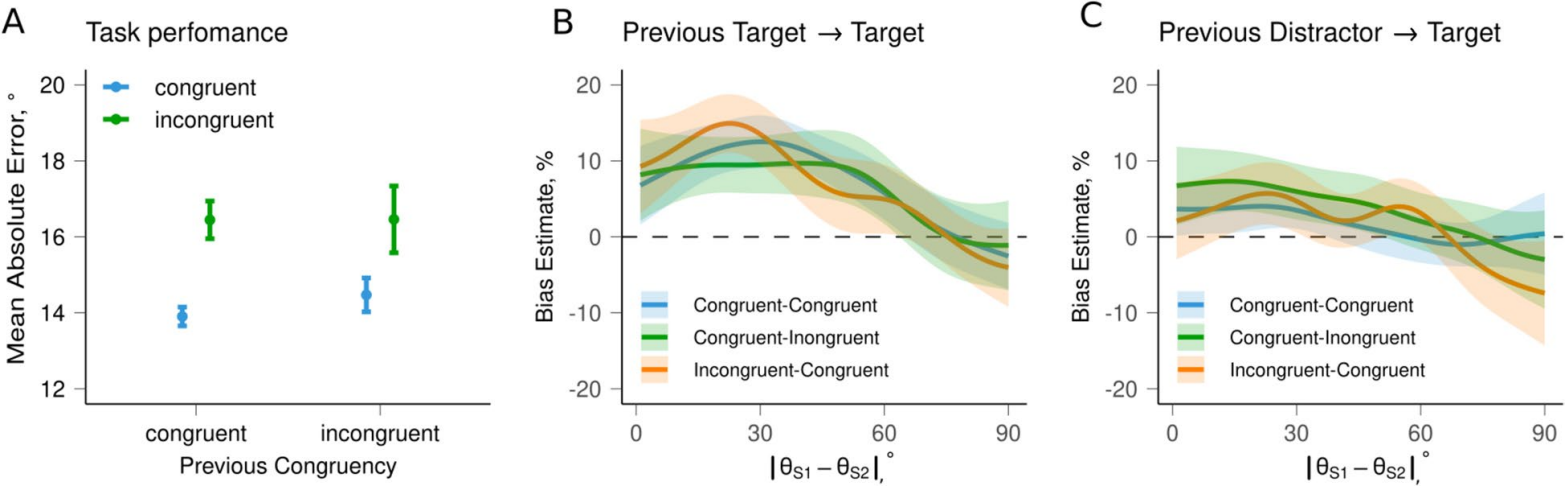
Results of Experiment 3 (*N* = 36 participants,* N*_trials_ = 720). **A** Overall performance. Mean absolute error (bars) with 95% confidence intervals (error bars) for each condition. **B**, **C** Serial dependence as a function of the angular difference between consecutive targets (**B**) or between the previous distractor and the current target (**C**). Lines show the mean bias; shaded regions show 95% confidence intervals

Performance was better in the congruent condition (*M* = 14.05, *SD* = 15.36) compared to the incongruent condition (*M* = 16.45, *SD* = 17.75; *F*(1,35) = 10.82, *p* = 0.002), demonstrating the effectiveness of congruency manipulation (see Fig. 8A). There were no significant differences found for previous congruency (*F*(1,35) = 0.19, *p* = 0.669) or their interaction (*F*(1,35) = 0.03, *p* = 0.871).

#### Serial dependence effect

The analysis revealed SD for two sequential targets across conditions (*M* = 0.07, *SD* = 0.12, *t*(35) = 8.76, *p* < 0.001). A two-way repeated measures ANOVA at each step of angular differences between the current and previous targets did not show significant differences between conditions (Fig. 8B). The ANOVA on the mean bias values also did not show a significant effect of condition (*F*(2, 70) = 0.03, *p* = 0.961). The results of the Bayesian ANOVA on the average bias computed for each participant across dissimilarity levels also supported the baseline model (*BF*_10_ = 0.09 ± 1.75%) over models that include the effects of congruency and previous congruency, indicating that the null hypothesis is more likely than the alternative. In an exploratory analysis of “peak” locations for SD curves within the orientation dissimilarity space for each participant and condition, Bayesian ANOVA did not show an effect of condition on peak locations, although the test results were inconclusive (*BF*_10_ = 1.28 ± 0.75%). Taken together, the results suggest a lack of the congruency effect on SD.

As in Experiment 2, we also examined in Experiment 3 whether SD from previous distractors differs from that of previous targets. We did observe a significant SD of previous distractors on the current target (*M* = 0.02, *SD* = 0.11, *t*(35) = 3.02, *p* = 0.003), although the magnitude was smaller than the SD effect from the previous target. However, once again, we found no differences between the conditions on the difference between conditions for each 1-degree step of similarity (see Fig. 8C). The ANOVA on the mean values found no significant effect of condition (*F*(2, 70) = 1.35, *p* = 0.265). In terms of differences in SD from non-reported items compared to reported ones, a paired-samples t-test showed that SD was significantly stronger for the previous target than for the previous distractor (*t*(35) = 4.18, *p* < 0.001). Overall, these findings indicate that probabilistic cueing has no influence on SD, as long as the factor of whether an item was reported or not is controlled for.

## Discussion

We conducted three experiments to investigate how the prioritization in VWM content affects the magnitude of serial dependence (SD). In Experiment 1, we manipulated prioritization by instructing participants to make extra effort to hold information in memory as it would be needed for a later report. This manipulation increased SD towards the memorized stimulus: active memory maintenance of the previous target led to a stronger bias in the current response. Within streaks, SD was observed only when the first stimulus was cued, with attractive biases strongest at an angular difference ∼35°. Across streaks, SD from the previous streak’s Stimulus 2 influenced the current streak’s Stimulus 1, and this bias persisted even in delayed responses, suggesting that prioritized representations can still be influenced by prior stimuli under specific interference conditions. In Experiments 2 and 3, we manipulated attentional prioritization through pre-cueing or post-cueing. Despite clear changes in memory fidelity, we found no significant differences in bias strength between congruent and incongruent conditions, suggesting that attentional prioritization through pre-cueing or post-cueing in a two-stimuli setup does not impact SD.

### Does prioritization affect serial dependence strength?

We found that prioritization of the inducer leads to stronger SD when manipulated via instruction to keep an item in VWM, but not via simple attentional cueing. The lack of effect from prioritization through attention diverges from the findings from the seminal paper by Fischer and Whitney [21] and the later studies that reported a significant impact of attention on SD ([18, 19, 23, 32, 48, 49], see [39], for a review). There are two complementary explanations for the discrepant results in the current study (Exp. 1 vs. Exps. 2 and 3) and when compared to the previous literature.

First, previous results reporting the effect of attention on SD can be explained by a confounding influence of reports. When Fischer and Whitney [21] reported the gating of serial dependence by attention, they asked observers to report the orientation of a Gabor on a cued location and found that the cued and reported item creates serial dependence while the non-cued one does not (even if it is at the same location as the currently tested item). Similarly, when Fritsche and de Lange [23] analyzed the effect of attending to the size or orientation of a Gabor patch on the orientation report of a subsequent stimulus, they manipulated whether the size or orientation of an inducer should be reported. Czoschke et al. [18] and Fischer et al. [19] used post-cueing to determine which stimulus is reported and found that only cued items created serial dependence. However, the cued stimulus was also the one reported (or, in the case of Exp. 2 in [18], the observers prepared to report the cued stimulus even though the report was omitted in 25% of trials). Notably, our results from Exps. 2 and 3 confirm these findings by showing that serial dependence from non-reported items was absent with pre-cueing (Exp. 2) and diminished with post-cueing (Exp. 3). In contrast, when we compared reported and cued against reported but not cued items, we do not find any difference. Similarly, non-reported items have equally weak (Exp. 3) or absent (Exp. 2) SD regardless of whether they are prioritized. The weak SD in one case is expected, as Exp. 3 — unlike Exp. 2, which used pre-cueing — employed a post-cue, meaning prioritization occurred after the perceptual stage. Thus, we suggest that the findings of previous studies can be better explained by the need to report an attended item or feature and not by the attention itself.

Second, the discrepancy between prioritization effects in Exp. 1 and Exps. 2 and 3 suggests that the effect of prioritization on SD might depend on the nature of prioritization itself. Specifically, in Exp. 1, prioritized items were to be maintained in memory even after they were reported. In Exps. 2 and 3, both prioritized and non-prioritized items could have been held in memory up until response time, albeit with different precision. Thus, it is possible that in Experiment 1 serial dependence in cued trials combines the effect of the previous report with the effect of the currently maintained item, while in Exps. 2 and 3, SD only reflects the effect of the previous report (perhaps with an additive effect of the previously reported stimuli). In short, maintaining the item in memory may influence subsequent processing, whereas once it is discarded after the report, prioritization becomes irrelevant.

Our findings also suggest that prioritization does not consistently protect against SD, as SD also occurred when the current item was prioritized with an additional memory requirement (Experiment 1) or with an attentional cue (Experiments 2 and 3). Results of Experiment 1 further showed significant interactions between the cueing of previous and current streaks for the bias from Stimulus 2 of the previous streak on Response 1 of the current streak. Specifically, SD was observed in three out of four conditions, with the bias absent for non-cued current trials when the previous streak was cued. While this could be due to the presence of additional interference from a maintained representation of a previous Stimulus 1 and its corresponding report (Response 3), SD still occurred when the current trial was cued, regardless of cueing in the previous streak. The results of Experiments 2 and 3 confirmed this finding, as attentionally cueing of the current item did not reduce the bias from the previous item (irrespective of whether the previous item was cued). In other words, prioritizing the current stimulus does not seem to protect against bias.

Overall, our findings that active memory maintenance intensifies SD align with prior results showing that focusing on memorizing past stimuli can enhance attraction effects toward them (e.g., [18, 19]). However, we highlight that it is the memory maintenance and not attention that modulates the bias strength, against the conclusions of the recent reviews [17, 39, 40, 46]. The effect of attention in previous studies is likely explained by the confounding influence of reports. Our results also diverge from studies proposing that giving more attention to the current stimulus offers better protection of items held in VWM from interference [4, 57]. The lack of protective effects or heightened vulnerability of cued items is consistent with Vergauwe et al. [59], who found that prioritizing visual memories does not consistently make them more vulnerable or resilient to perceptual interference. We observed partial protection only for information held in memory that had undergone decision-making (no SD from Stimulus 2 to Response 3 in Experiment 1). Nevertheless, it is also possible that the differences observed across experiments reflect not only variations in memory maintenance but also increased noise or uncertainty in the higher-load conditions. Therefore, the higher level of noise could make the more subtle effects of prioritization via attention difficult to detect. However, we consider this explanation less likely, as the Bayesian ANOVA supports the absence of a prioritization effect rather than insufficient sensitivity to detect it. In sum, we suggest that prioritization can affect SD if it leads to memory maintenance after the report, but not otherwise, and it creates no protection from SD or vulnerability to it.

### Does noise or uncertainty affect serial dependence strength?

More broadly, our study tested the idea that changes in noise in sensory or mnemonic signals, or the associated uncertainty, can affect SD strength. While this idea is intuitively appealing and supported by several previous studies (see [46], for a review), our findings present a more nuanced perspective. Specifically, decreasing the uncertainty about previous stimuli by instructing participants to maintain them in memory led to a stronger SD toward those memorized stimuli. In contrast, manipulating the level of noise through attentional prioritization via pre-cueing or post-cueing did not produce significant differences in bias strength.

Importantly, in the first case, we observed a stronger effect from changes in the previous stimulus noise, although the noise level of the current stimulus also contributed to SD strength. This stands in striking contrast to some previous studies (e.g., [11, 12, 26, 36]) reporting a stronger effect of current noise but little to no effect of previous noise. Notably, these studies manipulated external noise via factors such as contrast or spatial frequency of Gabor patches, while we focused on the internal noise. These differences suggest that, although noise level is indeed a key factor in modulating SD strength, attentional focus or uncertainty alone cannot fully explain the observed effects. Instead, a better understanding of the nature of noise — whether internal or external — is crucial, as additional factors likely influence whether the items in memory are protected from interference and thus contribute to SD strength.

### Comparison of results to model predictions

We modeled the predictions of the two models of serial dependence, the Bayesian model and the Demixing Model. Both models predicted that SD should become stronger when the inducer (i.e., the item on N-1 trial) is prioritized. The Bayesian model additionally predicted that SD should become weaker when the current item is prioritized. Exp. 1 but not Exps. 2 and 3 provide some support for the former prediction, but none of the experiments support the latter.

The Bayesian model predicted an attractive bias, with larger SD when the previous trial had a smaller noise level and smaller SD when the current trial had a smaller noise level. This partly aligns with our findings from Exp. 1: We did observe stronger SD when the previous trial was cued and hence had a smaller noise level (Fig. 4B). However, we did not observe the same effect in other experiments. Furthermore, none of the experiments showed a significantly smaller SD when the current trial had a smaller level of noise, and the previous one had a higher noise level as the model predicted (see Fig. 4D for the non-cued previous streak). Thus, we believe that the results of Exp. 1 cannot be taken as providing support for the Bayesian model, and overall, the support for its predictions is low.

The Demixing Model predictions are similar to the ones of the Bayesian model when it comes to the effect of the previous noise. Thus, the observed increase in SD as the noise of the previous item decreased in Exp. 1 is in line with the model’s predictions. However, the Demixing Model does not make linear predictions about the effect of the current noise (Fig. 2). The lack of the current item noise effect in this experiment is then not unexpected. What is unexpected from this model’s perspective is a lack of any significant differences in Exps. 2 and 3. Thus, similarly to the Bayesian model the current study does not support the Demixing Model predictions.

However, it is still possible that prioritization affects the noisiness of representations leading to the response but not the representation of the response. If the representation of the stimulus is discarded after the response (or transformed into a response-compatible template with fixed precision), then it explains why there is no effect of cueing on SD in Exps. 2 and 3. Exp. 1 then shows the effect of cueing because observers are explicitly asked to keep the representation in memory after responding. The lack of effect of cueing on the SD from non-reported items in Exps. 2 and 3 can be explained by the generally very weak or absent SD in these conditions. This hypothesis, however, does not explain the lack of effect of the current noise predicted by the Bayesian model.

In sum, while both the Bayesian and Demixing models can account for some aspects of our findings, they do not predict the lack of prioritization effects in Exps. 2 and 3. This highlights the need for refined models that better explain the empirical findings.

### The susceptibility to biases and the format of representations in VWM

Previous studies suggest that VWM prioritization could lead to different representational states: prioritized items are actively coded in VWM, while unprioritized items might rely on a different mechanism that does not require sustained neural activity [9, 34, 50, 62, 64, 65]. Consequently, the way information is maintained may shape its vulnerability to cognitive biases, potentially being more subject to interference, whereas actively coded items might be more resistant (“protection” hypothesis; [37, 58]). Alternatively, the active state might lead to higher susceptibility to biases (“vulnerability” hypothesis; [38]). Both of these ideas are not without contest, as some previous results suggest that an item’s prioritization in VWM does not affect its susceptibility to distraction [65] and that task-irrelevant (unprioritized) information from the previous trial is not maintained exclusively in an activity-silent manner [3].

Our results also provide a complicated picture. Speculatively, the absence of SD from Stimulus 2 to Response 3 in Experiment 1 suggests that actively coded information held in VWM may become less susceptible to external visual interference over time, highlighting the importance of the memory retention stage in explaining SD. However, we did not have a control condition where Stimulus 1 would be uncued but still reported twice, making it difficult to make any strong claims. We also found that the previous streak (Stimulus 2) influences the current one (both Response 1 and Response 3) only when the latter is prioritized, supporting the “vulnerability” hypothesis. At the same time, in Exps. 2 and 3 we did not find any effect of cueing, further complicating the matter.

One of the key points in this study is the effect of prioritization via memory maintenance. However, it raises an important question: at which stage does this prioritization influence the representation of stimuli? Does it affect encoding, or does it shape stimulus representation during the maintenance phase? Our findings suggest that the impact of prioritization emerges specifically during the maintenance stage. A stronger SD in the next trial — but not a weaker SD in the current trial — suggests that encoding remains unaffected by prioritization. Additionally, Experiments 2 and 3 showed no effect of prioritization, suggesting that the observed phenomenon is related to a late phase of maintenance rather than encoding.

## Conclusions

In summary, our findings from three experiments highlight the nuanced effects of prioritization of a representation in memory, visual interference, and their impact on SD. Our results both confirm and challenge prior research, revealing that active memory maintenance leads to stronger SD, suggesting a heightened bias inherent to these conditions beyond mere attentional effects. Additionally, the prioritization of the current stimuli does not always significantly influence its susceptibility to bias; rather, biases from previous streaks can persist even when current stimuli in VWM are prioritized. These insights contribute to a deeper understanding of how memory and attention shape perceptual judgments and biases in sequential decision-making tasks.

## Methods

### Power analyses

We conducted simulation-based power analysis for all experiments to determine the required sample sizes for detecting significant effects. Power was defined as the proportion of simulations where the null hypothesis was rejected. For each scenario, we simulated 1000 datasets with 18 participants each (18 was considered a minimum size based on the conventions in the field) and 648 trials for each participant, assuming a 1-degree difference in the magnitude of SD effect (based on the previous literature: [11, 12]). The noise and the baseline magnitude of SD in the observer’s responses were estimated based on Houborg et al. [29]. Subsequently, a statistical test was conducted for each simulated sample (t-test for Experiment 1 and repeated-measures ANOVA for Experiment 2 and 3, both at an alpha level of 0.05). Based on the results, a sample size of 18 participants was sufficient for detecting significant effects in both experiments (Experiment 1: power = 0.94,Experiment 2 and 3: power = 0.99). However, in the process of revising the paper, we doubled the original sample size for Experiments 2 and 3 to further enhance statistical power. The code used for the power analysis is available on the Open Science Framework (OSF) and can be accessed via the following link: https://osf.io/wunf8/?view_only=3ddc35f3c3ac4ffc8947a56200ffe4c5

### Experiment 1

#### Participants

Eighteen volunteers (10 women; *M*_age_ = 25.9 years, *SD*_age_ = 4.3 years) participated in the experiment in exchange for monetary compensation. All of them had normal or corrected-to-normal vision. The research protocol for this and the following studies was approved by the local Ethics Committee (Human Inspired Technology Research Centre—HIT, protocol number 2023_236R2). Prior to the experiment, all participants provided written informed consent and were informed about the general purpose of the study and the experimental procedures.

#### Stimuli, design, and procedure

The procedure consisted of two identical sessions conducted on separate days, each comprising 324 trials (for a total of 648 trials). The experiment began with 6 practice trials, followed by the main experimental phase, which included 9 blocks of 36 trials each. Instructions were displayed on the computer screen at the beginning of each session. Each session took approximately 1.5 h, with participants allowed to take breaks between blocks. Stimulus presentation and response collection were managed using PsychoPy software v.2023.2.0 [47], using an HP p1230 screen (85 Hz, 1920 × 1440 resolution). Participants were positioned approximately 60–65 cm away from the screen.

Each trial consisted of the presentation of a Gabor patch followed by an adjustment task (see Fig. 3). The Gabor patches had a diameter of 5.5° of visual angle (dva) and a frequency of 2 cycles/dva, with RGB (Red, Green, Blue) values of 0 and 255 for min and max luminance, respectively. They were displayed at the center of the computer screen against a gray background (RGB 128). The orientation of the Gabor patches was randomized and varied between 0 and 180°. To clarify the design in the following text, we use the term “streak” to describe two consecutively presented stimuli: the first Gabor patch (Stimulus 1) was either cued or non-cued, while the second (Stimulus 2) was always non-cued (see below).

The experiment included two conditions: a standard report condition (67% of streaks) and a report-and-hold-in-memory condition (33% of streaks). The two conditions were randomly interleaved.

In the standard report condition, we instructed participants to report the orientation of each Gabor patch immediately after its presentation (respectively Responses 1 and 2). Throughout all intervals, a fixation point (a white cross with a size of 0.05 dva) appeared at the center of the screen. The streak started with a 2000 ms fixation cross, followed by the first Gabor patch displayed for 500 ms. Then, a 2000 ms fixation cross was presented. Participants then had 4500 ms to complete the adjustment task, in which they saw a circle with a diameter of 2 dva containing a bar that they had to rotate to match the orientation of the Gabor patch using the right and left arrow keys. Pressing the spacebar confirmed their response. After another 1000 ms fixation cross, we presented the second Gabor patch for 500 ms, followed by a 2000 ms fixation cross and a second response period of 4500 ms.

In the report-and-hold-in-memory condition, participants additionally saw a cue (red circle, 1.2 dva diameter, 1000 ms) before the first Gabor was displayed (Fig. 3). This cue indicated that they would need to report the orientation of the first Gabor twice: once immediately after its presentation (Response 1) and again (Response 3) after reporting the orientation of the second Gabor (Response 2), with a 1000 ms pause in between. During Response 3, an additional instruction reminded participants that they needed to report the orientation of the cued stimulus. This task required participants to retain the first stimulus in memory while perceiving and reporting the second stimulus.

Throughout the experiment, participants were instructed to fixate on the center of the screen and report the orientation of each stimulus as accurately as possible.

### Experiment 2

#### Participants

Thirty-six participants (12 women; *M*_age_ = 26.8 years, *SD*_age_ = 4.9 years) took part in Experiment 2 (the number of participants before exclusion was fifty-two; sixteen participants were excluded due to low accuracy, defined as a circular standard deviation of response errors greater than 30°, and replaced with new ones). The study was conducted online through the Prolific platform in exchange for a monetary reward. All participants had normal or corrected-to-normal vision and provided online informed consent before participating in the study.

#### Stimuli, design, and procedure

A credit card adjustment procedure was used to control the size of the visual stimuli in this online experiment [35]. Participants were instructed to position themselves at a distance of approximately 60–65 cm from the computer screen.

The experiment was conducted in a single session consisting of 720 trials. It began with a practice part of 12 trials, followed by the main experimental part divided into 10 blocks of 72 trials each. The entire procedure lasted approximately 1.5 h, with participants allowed to take breaks between blocks. The experiment was developed using PsychoPy software v.2023.2.0, and responses were collected via the online platform Pavlovia.org [47].

Each trial began with a white fixation cross, sized at 0.05 dva, at the center of the screen, which remained present throughout the experiment. Participants fixated on this cross for 1000 ms before a red pre-cue circle, with a diameter of 0.8 dva, randomly appeared on either the left or right side of the screen for another 1000 ms. This pre-cue indicated which of the upcoming Gabor patches participants needed to memorize. After the pre-cue, two Gabor patches (diameter 3.3 dva, spatial frequency 8 cycles/dva; min and max RGB values of the Gabor patches 0 and 255, respectively) were simultaneously displayed for 1000 ms on both sides of the screen, centered 4 dva from the screen’s center. One Gabor patch served as the target and the other as a non-target, with their orientations independently randomized between 0 and 180°. The stimuli were presented against a gray background (128 RGB).

The study design consisted of two conditions: a congruent condition (75% of trials) and an incongruent condition (25% of trials). We instructed participants to report the orientation of the cued Gabor patch. In the congruent condition, the adjustment bar subsequently appeared on the same side as the cued Gabor patch, and participants had to report the orientation of that Gabor. In the incongruent condition, the adjustment bar subsequently appeared on the opposite side of the cued Gabor patch, and participants had to report the orientation of the non-cued Gabor (e.g., if the right Gabor was cued, but they were asked to report the orientation of the left Gabor, see Fig. 5). Throughout the experiment, we instructed participants to maintain their gaze on a fixation cross at the center of the screen and report the stimulus orientation as accurately as possible. In each trial, congruent and incongruent conditions were selected using a weighted random process, with congruent conditions occurring three times more frequently. In contrast to Experiment 1, no predefined streaks were imposed.

### Experiment 3

#### Participants

Thirty-six participants (12 women; *M*_age_ = 26.1 years, *SD*_age_ = 4.3) took part in Experiment 3 online through the Prolific platform in exchange for a monetary reward (the number of participants before exclusion was fifty-three; seventeen participants were excluded due to low accuracy based on the same criteria used in Experiment 2 and replaced with new ones). All participants had normal or corrected-to-normal vision and provided online informed consent before participating.

#### Stimuli, design, and procedure

The design of Experiment 3 was identical to Experiment 2, with the key difference being the use of a post-cue instead of a pre-cue.

The post-cue appeared 500 ms after the stimuli presentation and lasted for 100 ms. After an additional 400 ms, the adjustment task began. After the task was completed, there was an inter-trial interval of 3000 ms. This timing was designed to maintain a 1000 ms pause between stimuli and response, and a 3000 ms interval between the response and the next stimulus, in accordance with the timing used in Experiment 2.

## Data Availability

All data generated or analysed during this study are included in this published article, its supplementary information files and publicly available repositories Open Science Framework (OSF) https://osf.io/wunf8/?view_only=3ddc35f3c3ac4ffc8947a56200ffe4c5.

## Abbreviations

DM: Demixing Model
Dva: Degrees of visual angle
fMRI: Functional magnetic resonance imaging
MEG: Magnetoencephalography
RGB: Red, Green, Blue
SD: Serial dependence
VWM: Visual working memory
